# Pleural abrasion versus apical pleurectomy for primary spontaneous pneumothorax: a systematic review and Meta-analysis

**DOI:** 10.1186/s13019-023-02207-3

**Published:** 2023-04-06

**Authors:** Jaewon Chang, Vignesh Ratnaraj, Vincent Fu, Michael Jiang, Varun Peri, Minhtuan Nguyenhuy, Phillip Antippa

**Affiliations:** 1 St George Hospital, Kogarah, Sydney, NSW 2217 Australia; 2 The Royal Melbourne Hospital, Parkville, Melbourne, VIC 3050 Australia; 3 The University of Melbourne, Parkville, Melbourne, VIC 3050 Australia; 4 Austin Hospital, 3084 Heidelberg, Melbourne, VIC Australia; 5 Western Health, 3011 Footscray, Melbourne, VIC Australia

**Keywords:** Primary spontaneous pneumothorax, Pleurodesis, Pleural abrasion, Pleurectomy

## Abstract

**Background:**

Surgical approach is the most effective treatment for primary spontaneous pneumothorax. The two most widely adopted surgical methods are mechanical abrasion and apical pleurectomy, in addition to bullectomy. We performed a systematic review and meta-analysis to examine which technique is superior in treating primary spontaneous pneumothorax.

**Methods:**

PubMed, MEDLINE and EMBASE databases were searched for studies published between January 2000 to September 2022 comparing mechanical abrasion and apical pleurectomy for treatment of primary spontaneous pneumothorax. The primary outcome was pneumothorax recurrence. Secondary outcomes included post-operative chest tube duration, hospital length of stay, operative time and intra-operative of blood loss.

**Results:**

Eight studies were eligible for inclusion involving 1,613 patients. There was no difference in the rate of pneumothorax recurrence between pleural abrasion and pleurectomy (RR: 1.34; 95% CI: 0.94 to 1.92). However, pleural abrasion led to shorter hospital length of stay (MD: -0.25; 95% CI: -0.51 to 0.00), post-operative chest tube duration (MD: -0.30; 95% CI: -0.56 to -0.03), operative time (MD: -13.00; 95% CI -15.07 to 10.92) and less surgical blood loss (MD: -17.77; 95% CI: -24.36 to -11.18).

**Conclusion:**

Pleural abrasion leads to less perioperative patient burden and shorter hospital length of stay without compromising the rate of pneumothorax recurrence when compared to pleurectomy. Thus, pleural abrasion is a reasonable first choice surgical procedure for management of primary spontaneous pneumothorax.

## Introduction

Primary Spontaneous Pneumothorax (PSP) is an abnormal collection of air in the pleural space, occurring predominantly in young and otherwise healthy individuals without clinically apparent lung pathology [1]. It is one of the most common thoracic diseases of the young, with an estimated annual incidence of up to 22.7 per 100,000 people [2, 3]. The disease displays a male preponderance of 1:3.3 and cigarette smoking as a major risk factor [3, 4]. While pneumothoraces can be managed successfully with a variety of techniques ranging from observation to surgical techniques, spontaneous pneumothoraces without surgical treatment are characterized by their tendency to recur at a risk of approximately 30% at one year, thus producing a significant burden of disease [5].

A variety of approaches to manage PSP exist, including conservative therapy, drainage of air via needle aspiration or chest drain insertion, and definitive surgical intervention. Surgical procedures performed with video-assisted thoracoscopy include a combination of bullectomy, mechanical abrasion, pleurectomy, chemical pleurodesis (notably talcum powder) [6, 7]. The two main objectives of surgical approach are resection of bleb or bullae [8] and obliteration of the pleural space to achieve pleural symphysis to prevent recurrence of pneumothorax [9]. Generally, surgical interventions are reserved for recurrent pneumothorax, bilateral pneumothorax, persistent air leak despite chest tube drainage or failure of lung expansion, spontaneous haemothorax, pregnancy and professions at risk, such as divers and pilots [9].

Current evidence regarding superiority of bullectomy with pleural abrasion versus pleurectomy for PSP is lacking. As such, even authoritative guidelines [9, 10] are ambiguous in providing a gold standard intervention for PSPs.

The aim of this systematic review and meta-analysis is to compare the outcomes between mechanical abrasion and apical pleurectomy in patients with primary spontaneous pneumothorax. This comparison is important to guide clinical decision making, reduce heterogeneity in surgical management and to understand what clinically relevant outcomes are associated with each procedure.

## Materials and methods

### Literature search strategy

Literature search was conducted on the PubMed, Ovid MEDLINE and EMBASE databases from 1st January 2000 to 3rd September 2022. Keywords and MeSH terms relating to “pneumothorax” in combination with “pleurodesis” and “pleurectomy” were used in the search strategy to capture the relevant literature. PRISMA (Preferred Reporting Items for Systematic Reviews and Meta-Analyses) guidelines were adhered to.

**Fig. 1:**
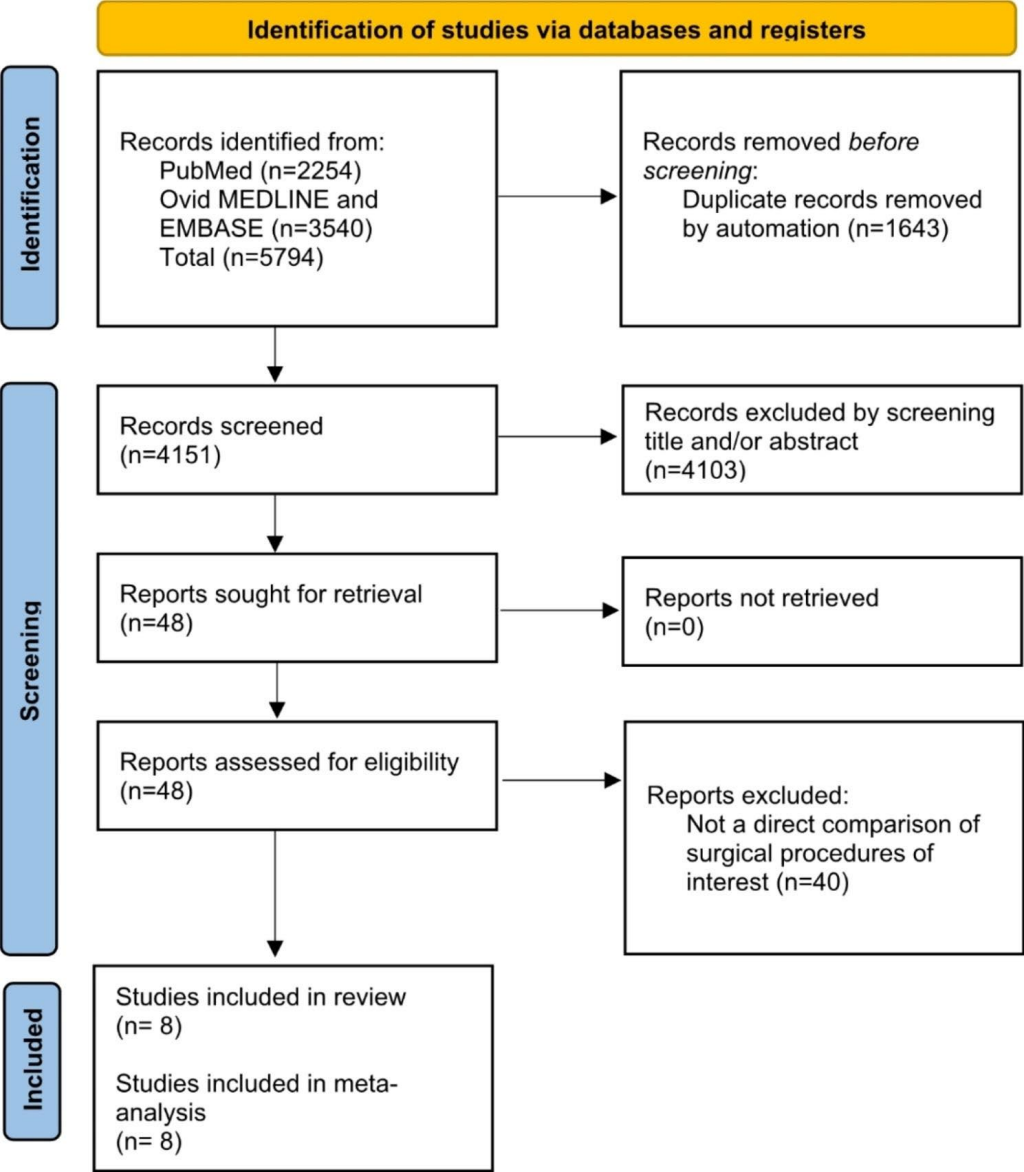
PRIMSA flowchart

Two authors (JC and VF) conducted independent literature searches on databases to identify eligible studies. Screening of title, abstract and full manuscript were performed individually. Forty-eight full manuscripts were reviewed independently for eligibility. Authors’ discrepancies in manuscript eligibility were resolved through consensus or referral to third author (VR). Details are outlined in Fig. 1.

### Eligibility criteria

The eligibility criteria for study inclusion were: [1] any retrospective or prospective investigative studies excluding case reports; [2] primary spontaneous pneumothorax defined as pneumothorax occurring in patients with otherwise healthy lung; [3] treatment including pleural abrasion and pleurectomy; [4] presentation of surgical outcomes stratified by surgical procedure; [5] publication date from January 2000 to search date; and [6] English language publication.

The primary surgical outcome of interest was pneumothorax recurrence. Secondary outcomes included post-operative chest tube duration, hospital length of stay, operative time and intraoperative blood loss.

### Risk of bias

Quality assessment for non-randomised studies was performed independently by two authors (JC and VF) using the Newcastle-Ottawa Scale [11] (Table 3). This scale assesses selection, comparability and outcome for quality and risk of bias.

**Table 1:** Summary of Study Characteristics

Author, Year	Patients (N, %)	Location	Study Design	Follow-up Duration and Method
Ayed 2000	N = 72 Abrasion = 39 (54%) Pleurectomy = 33 (46%)	Kuwait	Prospective Cohort	Follow-up 42 months (36–54) Hospital Database
Brophy 2021	N = 78 Abrasion = 61 (78%) Pleurectomy = 17 (22%)	Canada	Retrospective Cohort	Follow-up duration NR National and Local Database
Chang 2006	N = 65 Abrasion = 35 (54%) Pleurectomy = 30 (46%)	Taiwan	Retrospective Cohort	Median follow-up 25.4 months Hospital Database
Ocakcioglu 2019	N = 88 Abrasion = 48 (55%) Pleurectomy = 40 (45%)	Turkey	Retrospective Cohort	Mean follow-up 19.3 months Hospital Database
Owen 2022	N = 968 Abrasion = 791 (82%) Pleurectomy = 177 (18%)	USA	Retrospective Cohort	Follow-up 5 years (mean/median NR) Hospital Database
Patterson 2021	N = 52 Abrasion = 25 (48%) Pleurectomy = 27 (52%)	USA	Retrospective Cohort	Follow-up duration NR Hospital Database
Rena 2008	N = 220 Abrasion = 112 (51%) Pleurectomy = 108 (49%)	Italy	Prospective Cohort	Mean follow-up 46 months Hospital Database
Tuluce 2022	N = 70 Abrasion = 30 (43%) Pleurectomy = 40 (57%)	Turkey	Retrospective Cohort	Follow-up duration NR Hospital Database

PSP = primary spontaneous pneumothorax; NR = Not Reported

**Table 2:** Summary of baseline patient characteristics

Author, Year	Patient demographics (abrasion / pleurectomy, p-value where reported or total if not separated)
Ayed 2000	Age 25 ± 6; Female 7%; Laterality (L) 30.6%; smoker NR; VATS 100%;
Brophy 2021	Age 27.2/26.9; Female 33/18% Laterality (L) NR; smoker 43/47%; VATS 100/94%;
Chang 2006	Age 24.2/27.5,p = 0.165; Female 6/10%,p = 0.655; Laterality (L) 63/43%, p = 0.140; smoker 37/37%,p = 1.000; VATS 100%
Ocakcioglu 2019	Age 23.35/24.51,p = 0.73; Female 20.8/17.5%,p = 0.69; Laterality (L) 52.0/52.5%,p = 0.96; smoker 29.2/37.5%,p = 0.4; VATS 100%
Owen 2022	Age 19/19; Female 18.2/23.2%; Laterality (L) NR; smoker NR; VATS NR
Patterson 2021	Age 16.4/16.6; Female 12.0/19.2; Laterality (L) 56/70.4%; smoker 20/7%; VATS 100%;
Rena 2008	Age 24.5/25,p = 0.58; Female 20.7/16.7%,p = 0.58; Laterality (L) 44.7/41.7%,p = 0.75; smoker 56.7/50.5%,p = 0.46; VATS 100%
Tuluce 2022	Age 24.8 ± 6.8; Female 12.1%; Laterality (L) 40.9%; smoker 73.2%; VATS 93%

VATS = Video-Assisted Thoracoscopic Surgery

**Table 3:** Newcastle-Ottawa Scale

Author, Year	Selection				Comparability	Outcomes			Total /9
	Representativeness of the exposed cohort	Selection of non-exposed cohort	Ascertainment of exposure	Outcome of interest not present at the start of the study		Assessment of outcomes	Length of follow-up	Adequacy of follow-up	
Ayed 2000	↔	↔	↔	↔	↔↔	↔	↔	↔	9
Brophy 2021	↔	↔	↔	↔	↔↔	↔	↔	↔	9
Chang 2006	↔	↔	↔	↔	↔↔	↔	↔	↔	9
Ocakcioglu 2019	↔	↔	↔	↔	↔↔	↔	↔	↔	9
Owen 2022	↔	↔	↔	↔	↔↔	↔	↔	↔	9
Patterson 2021	↔	↔	↔	↔	↔↔	↔	↔	↔	9
Rena 2008	↔	↔	↔	↔	↔↔	↔	↔		8
Tuluce 2022	↔	↔	↔	↔	↔↔	↔	↔	↔	9

### Statistical analysis

Continuous variables were presented as either mean and standard deviation or median with interquartile range. Categorical variables were presented as numbers and/or percentages. Meta-analysis was performed using raw data presented in each study and summarised in the form of risk ratios (RR) for binary outcomes and mean difference (MD) for continuous outcomes. Outcomes of interest reported as median and interquartile range were converted to mean and standard deviation by the method outlined by Luo et al. [12], assuming a normal distribution for the cohort. The fixed effects model was used to assess effect estimates. Tau^2^ (Τ^2^) and I^2^ values were used to assess heterogeneity. I^2^ cut-off of 25%, 50% and 75% were used to indicate low, moderate, and high heterogeneity, respectively. Statistical significance was defined as p < 0.05. All statistical analysis was performed using Review Manager 5.4 (Cochrane Collaboration, Software Update, Oxford, UK).

## Results

### Study characteristics

The literature search returned 5,794 records for screening. After duplicates were removed, 4151 records were screened based on title and abstract. Forty-eight studies underwent full text review and application of inclusion criteria, following which eight studies were deemed eligible for inclusion in this review. Two studies were prospective [13, 14], and remaining six studies [15–20] were retrospective in nature. All eight studies were included in the meta-analysis [12–19].

The total study population was 1,613 and cohort sizes ranged from 52 to 968. All included studies directly compared bullectomy plus mechanical pleurodesis with bullectomy plus apical pleurectomy. A total of 1,141 patients underwent mechanical abrasion compared to 472 patients who underwent apical pleurectomy. Mean follow-up duration was greater than 12 months for five out of eight studies [13, 14, 16–18]. The baseline study characteristics are summarized in Tables 1 and 2.

### Surgical technique

Bullectomy was routinely performed in all studies. Where no blebs were detected, an apical wedge resection was performed in the studies. Reported techniques for pleurectomy were electrocautery and stripping [15, 16], blunt stripping [17, 19] and unspecified in the remaining four studies [13, 14, 18, 20]. The borders of pleural stripping were the upper margin of the fifth rib inferiorly, sympathetic trunk posteriorly, internal mammary artery anteriorly and to the height of the left subclavian artery on the left or brachiocephalic trunk on the right [14, 16, 17]. Commonly utilized materials for pleural abrasion were cautery scratch pad [15, 16, 19], gauze or mesh [13, 14, 17] and was unspecified in two studies [18, 20].

### Cohort demographics

No statistically significant baseline patient demographic differences were demonstrated between the mechanical abrasion and apical pleurectomy groups. Specifically, when comparing pleurectomy group to the mechanical abrasion group, age (MD: -0.11; 95% CI: -0.53 to 0.32; p = 0.63), female sex (RR: 0.89; 95% CI: 0.70 to 1.14; p = 0.36), smoking history (RR: 0.89; 95% CI: 0.71 to 1.10; p = 0.28) and left sidedness of pneumothorax (RR: 1.05; 95% CI: 0.87 to 1.27; p = 0.62) did not differ significantly.

### Clinical outcomes

**Fig. 2:**
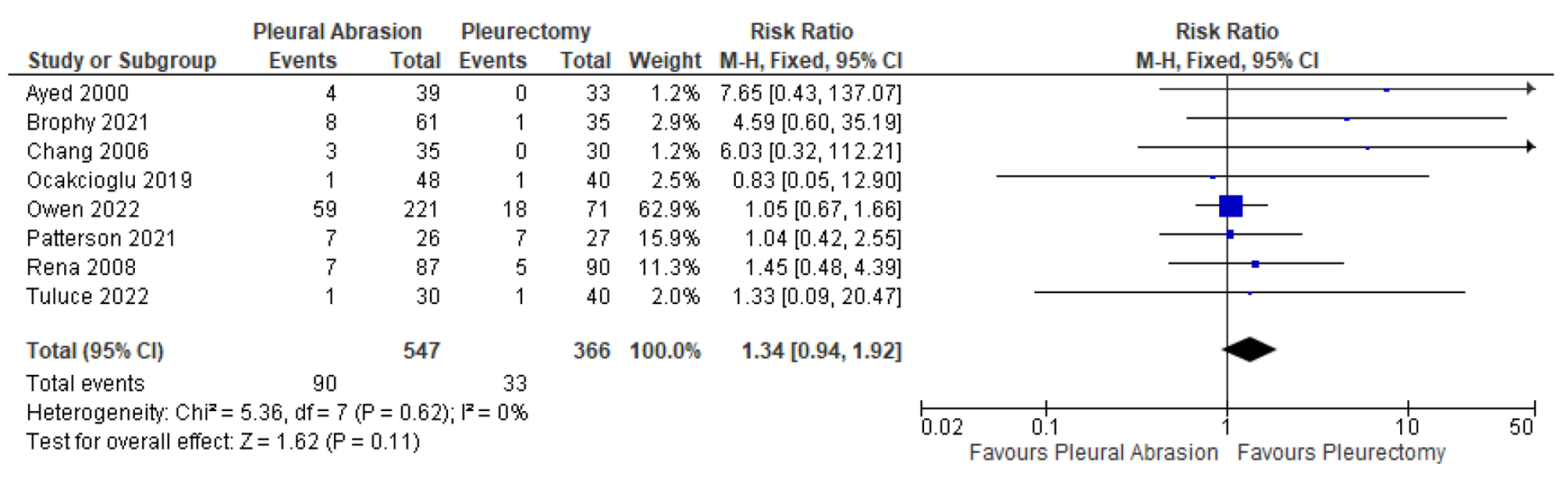
Forest plot displaying relative risk (RR) of pneumothorax recurrence rate post pleurodesis with pleural abrasion and pleurectomy

**Fig. 3:**
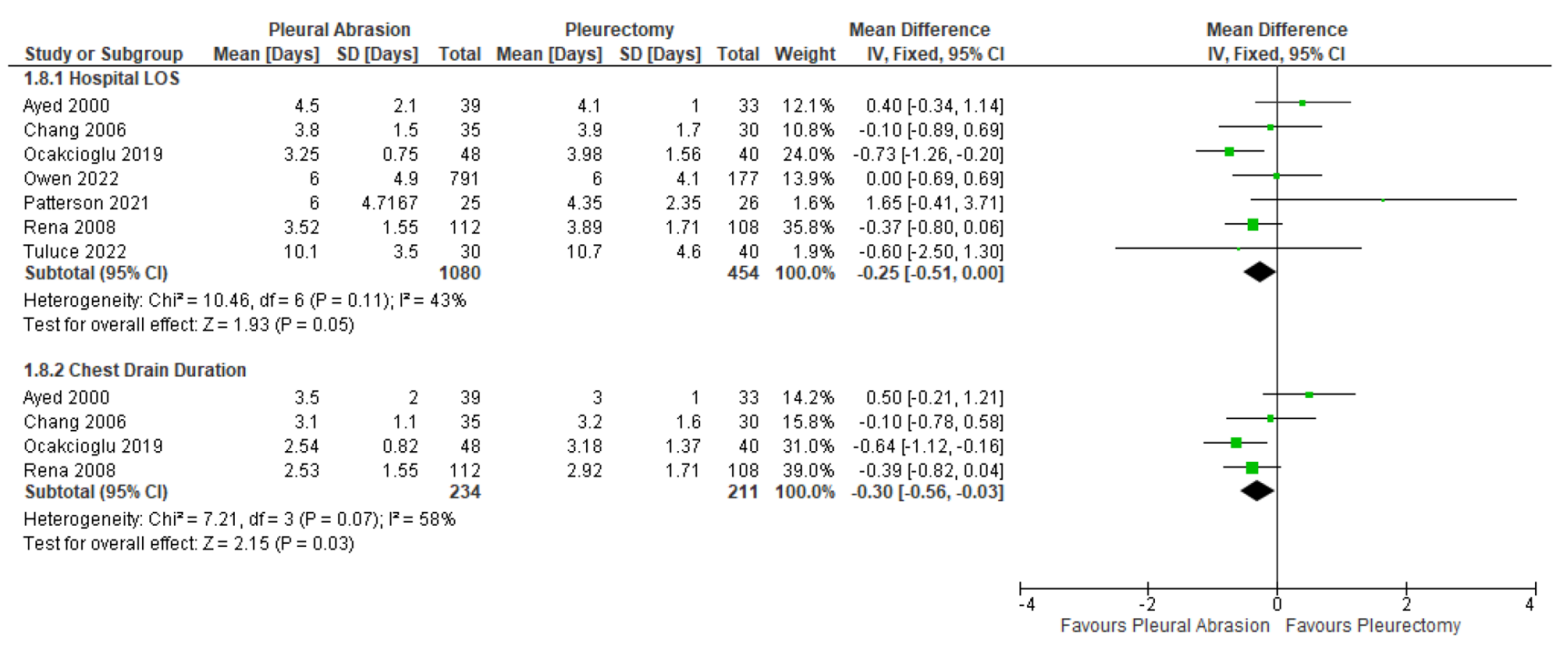
Forest plot displaying mean difference (MD) of mean hospital length of stay and chest tube duration between pleural abrasion and pleurectomy

**Fig. 4:**
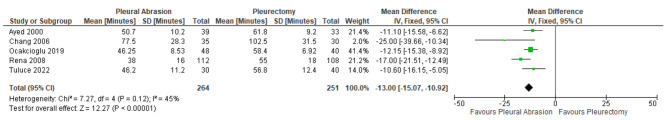
Forest plot displaying mean difference (MD) of mean operation time between pleural abrasion and pleurectomy

**Fig. 5:**
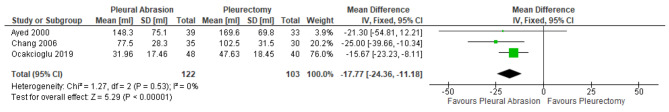
Forest plot displaying mean difference (MD) of mean blood loss between pleural abrasion and pleurectomy

Pneumothorax recurrence rate did not differ between surgical pleurodesis by mechanical abrasion compared to apical pleurectomy (RR: 1.34; 95% CI: 0.94 to 1.92; p = 0.11) (Fig. 2). Patients who underwent pleural abrasion had a statistically significant shorter hospital length of stay (MD: -0.25; 95% CI: -0.51 to 0.00; p = 0.05) and post-operative chest tube duration (MD: -0.30; 95% CI: -0.56 to -0.03; p = 0.03) (Fig. 3). Similarly, mechanical abrasion was associated with shorter operative time (MD: -13.00; 95% CI -15.07 to 10.92; p < 0.01) (Fig. 4) and less surgical blood loss (MD: -17.77; 95% CI: -24.36 to -11.18; p < 0.01) (Fig. 5).

Sensitivity analysis was performed by removing the study with largest weighting from each outcome. This resulted in a statistically significant association between pleural abrasion and pneumothorax recurrence when compared to pleurectomy (RR: 1.84, 95% CI: 1.02 to 3.29; p = 0.04). Pleural abrasion was no longer statistically significant for reduced hospital length of stay (MD: -0.19; 95% CI: -0.51 to 0.13; p = 0.25) and post-operative chest drain duration (MD: -0.23; 95% CI: -0.58 to 0.11; p = 0.18). Pleural abrasion was still associated with shorter operative time (MD: -13.59; 95% CI: -16.15 to -5.05; p < 0.01) and lower operative blood loss (MD: -24.41; 95% CI: -37.84 to -10.97; p < 0.01).

## Comment

To the authors’ knowledge, this is the first systematic review and meta-analysis to directly compare outcomes of mechanical pleural abrasion and apical pleurectomy for the treatment of primary spontaneous pneumothorax. Our meta-analysis demonstrated that there is no statistically significant difference between mechanical pleural abrasion and pleurectomy in pneumothorax recurrence in cohorts matched for potentially confounding factors including age, gender, smoking history and laterality of pneumothorax. In addition, pleural abrasion is associated with shorter hospital length of stay, post-operative chest tube duration, operative time and less surgical blood loss.

Hospital length of stay in all observed studies displays a strong correlation to chest tube duration, as by the time of chest drain removal, other important factors such as pain and mobility can be controlled, especially in a young cohort such as those with primary spontaneous pneumothorax. Several studies have demonstrated statistically indifferent levels of patient reported short-term pain and opioid prescription between mechanical abrasion and apical pleurectomy [14, 16, 18]. The factors that influence post-operative chest tube duration in pleurodesis are expansion of lung, absence of air leak and drain output [13]. Longer operative time associated with apical pleurectomy may contribute to slower complete re-expansion, and more surgical blood loss may be contributing to the lengthier chest tube duration observed in the apical pleurectomy group. Further, another important yet unclear parameter to be further examined is the clinical significance of increased blood loss in apical pleurectomy compared to mechanical abrasion, with some studies reporting no difference in blood transfusion requirements nor return to theatre [17, 19] where others found statistically significant increase in return to theatre in apical pleurectomy group [14].

Several studies have already demonstrated that surgical pleurodesis is superior to non-surgical managements in pneumothorax recurrence [2, 6, 21]. However, the choice of surgical procedure has traditionally been influenced by surgeon experience and preference, with no gold-standard directives published in reputable guidelines [8]. Our analysis demonstrated a trend towards apical pleurectomy for the prevention of recurrent pneumothorax, albeit without statistical significance. Whilst this trend may support older literature advocating for various degrees of pleurectomy in patients with spontaneous pneumothoraces [22], a clear advantage of apical pleurectomy was not demonstrated. In the absence of a clear benefit of apical pleurectomy over pleural abrasion in preventing pneumothorax recurrence, it is reasonable to consider pleural abrasion in the first instance given the ancillary benefits reported above.

This study has several limitations. In this meta-analysis, the 2022 study by Owen et al. [18] had large weighting in several domains given their larger relative sample size. Sensitivity analysis performed to circumvent this issue led to variations in statistical significance, notably superiority of pleurectomy over pleural abrasion in preventing pneumothorax recurrence. In addition, while advantages in terms of shorter operative time and less intra-operative blood loss were retained, pleural abrasion was no longer advantageous in terms of hospital length of stay nor post-operative chest drain duration. We believe this variation in results reflects a necessity for further research in this area, and that the true differences are likely to be small. Another limitation was the heterogeneity in follow-up duration of the studies. Pneumothorax recurrence is perhaps the most important measure in determining management strategies, and to have been able to stratify recurrence rates based on time after surgery would have provided a useful insight. Furthermore, owing to the small number of studies that were eligible for this systematic review and meta-analysis, we were limited in the outcomes that were able to be examined. Future higher-powered trials comparing mechanical pleural abrasion to apical pleurectomy are required before the superiority of one technique over the other can be confirmed.

## Conclusion

Our analysis demonstrates that there is no statistically significant difference in the rate of pneumothorax recurrence between bullectomy with pleural abrasion and apical pleurectomy. Pleural abrasion, however, leads to shorter hospital length of stay, post-operative chest drain duration, operative time and less surgical blood loss. Therefore, pleural abrasion is a reasonable first choice procedure for surgical management of primary spontaneous pneumothorax.

## Data Availability

All datasets will be made available on request to corresponding author via email.

## List of Abbreviations

PSP: Primary Spontaneous Pneumothorax
PRISMA: Preferred Reporting Items for Systematic reviews and Meta-Analysis
RR: Risk Ratio
MD: Mean Difference
CI: Confidence Interval
NR: Not Reported
VATS: Video Assisted Thoracoscopic Surgery
